# Characterization of SiO_2_ Plasma Etching with Perfluorocarbon (C_4_F_8_ and C_6_F_6_) and Hydrofluorocarbon (CHF_3_ and C_4_H_2_F_6_) Precursors for the Greenhouse Gas Emissions Reduction

**DOI:** 10.3390/ma16165624

**Published:** 2023-08-14

**Authors:** Minsu Choi, Youngseok Lee, Yebin You, Chulhee Cho, Wonnyoung Jeong, Inho Seong, Byeongyeop Choi, Sijun Kim, Youbin Seol, Shinjae You, Geun Young Yeom

**Affiliations:** 1 Department of Physics, Chungnam National University, Daejeon 34134, Republic of Korea; bss125576@naver.com (M.C.); dpqls5029@gmail.com (Y.Y.); paulati@naver.com (C.C.); jwn1748@gmail.com (W.J.); showing123@naver.com (I.S.); chlqudduq050@naver.com (B.C.); sjyou@cnu.ac.kr (S.Y.); 2 Institute of Quantum Systems (IQS), Chungnam National University, Daejeon 34134, Republic of Korea; kim_sijun@naver.com (S.K.); youbin0621@cnu.ac.kr (Y.S.); 3 Department of Advanced Materials Science and Engineering, Sungkyunkwan University, Suwon 16419, Republic of Korea; gyyeom@skku.edu; 4 SKKU Advanced Institute of Nano Technology (SAINT), Sungkyunkwan University, Suwon 16419, Republic of Korea

**Keywords:** plasma processing, greenhouse effect, greenhouse gas, global warming potential, alternative precursors, C_4_F_8_, C_6_F_6_, CHF_3_, C_4_H_2_F_6_, plasma etching, plasma diagnostics

## Abstract

This paper proposes the use of environmentally friendly alternatives, C_6_F_6_ and C_4_H_2_F_6_, as perfluorocarbon (PFC) and hydrofluorocarbon (HFC) precursors, respectively, for SiO_2_ plasma etching, instead of conventional precursors C_4_F_8_ and CHF_3_. The study employs scanning electron microscopy for etch profile analysis and quadrupole mass spectrometry for plasma diagnosis. Ion bombardment energy at the etching conditions is determined through self-bias voltage measurements, while densities of radical species are obtained using quadrupole mass spectroscopy. The obtained results compare the etch performance, including etch rate and selectivity, between C_4_F_8_ and C_6_F_6_, as well as between CHF_3_ and C_4_H_2_F_6_. Furthermore, greenhouse gas (GHG) emissions are evaluated using a million metric ton of carbon dioxide equivalent, indicating significantly lower emissions when replacing conventional precursors with the proposed alternatives. The results suggest that a significant GHG emissions reduction can be achieved from the investigated alternatives without a deterioration in SiO_2_ etching characteristics. This research contributes to the development of alternative precursors for reducing global warming impacts.

## 1. Introduction

The semiconductor industry has extensively embraced plasma technology for semiconductor device manufacturing [1,2]. In the etching process, plasma has become crucial for nanoscale material processing, enabling precise and deep etch profiles to meet the requirements of scaling down of transistor feature sizes [3]. As a results, plasma processing has become indispensable for the production of next-generation semiconductor devices, including high-aspect-ratio contact (HARC) hole.

Plasma etching utilizes various gases or gas mixture tailored to the target materials, such as semiconductors [4,5], metals [6,7,8], and dielectrics [9,10,11,12]. For dielectrics, such as SiO_2_ and Si_3_N_4_, fluorocarbon-based molecules, such as C_4_F_8_ and CHF_3,_ are widely adopted due to their polymerizing properties, which are particularly advantageous for achieving anisotropic etch profiles. During the etching process, fluorocarbon films passivate the sidewalls of the dielectric trench, preventing them from reacting with reactive etchants. However, the energetic plasma ion bombardment renders the fluorocarbon film unable to protect the trench bottom, resulting in an anisotropic etch profile [13,14,15]. Since anisotropy is a crucial characteristic in semiconductor manufacturing, large quantities of perfluorocarbon (PFC) and hydrofluorocarbon (HFC) precursors, such as C_4_F_8_ and CHF_3_, are extensively utilized in the semiconductor industry.

However, the utilization of the PFC and HFC precursors is subject to strict regulations due to their significant greenhouse effect [16,17]. Global warming potential (GWP) is a metric used to quantify the relative warming impact of greenhouse gases (GHGs) on the Earth’s climate system over a specified time horizon, typically, 20, 100, or 500 years, relatively to that of CO_2_ [18]. The GWP_100_ (GWP over a 100-year time horizon) of C_4_F_8_ and CHF_3_ is reported as 9540 and 12,400, respectively, indicating that these precursors have a greenhouse effect over 10,000 times stronger than CO_2_ [19]. Given the substantial global efforts to combat global warming in recent decades [20,21], the emission of these high-GWP gases is facing increasingly stringent restrictions in the industry [22].

In this context, researchers worldwide have been actively engaged in developing alternative plasma etching precursors that process low GWPs while demonstrating comparable etch performance to conventional ones. Sung et al. investigated the etching characteristics of SiO_2_ using the C_6_F_6_/Ar/O_2_ gas mixture in both capacitively coupled and inductively coupled plasma etching systems, comparing the etch results obtained from these different plasma systems [23]. Kim et al. studied SiO_2_ etching using C_4_H_3_F_7_O isomers and compared them with C_4_F_8_ through X-ray photoelectron spectroscopy, quadrupole mass spectroscopy, and scanning electron microscopy [24]. Furthermore, Lim et al. compared the etch results with a low-GWP precursor, C_6_F_12_O, to those with the conventional precursor, CF_4_ [25]. Although many papers, including the aforementioned ones, have documented the development of alternative low-GWP precursors in the field of semiconductor manufacturing [26,27,28,29,30], there are still numerous potential precursors that need to be investigated regarding their qualification as promising alternatives to conventional high-GPW precursors.

In this work, we propose the use of environmentally friendly alternatives, C_6_F_6_ and C_4_H_2_F_6_, as PFC and HFC precursors, respectively, for SiO_2_ plasma etching, in lieu of the conventional precursors C_4_F_8_ and CHF_3_, which are the most representative etching gases for dielectric etching [3]. C_4_F_8_ and CHF_3_ are selected as the reference precursors for PFC and HFC precursors, respectively, instead of other alternative low-GWP precursors, such as C_4_F_6_ (GWP < 1), to excavate additional alternatives that possibly show different etching performance from C_4_F_6_, which would provide a wide range of options to, for example, process engineers. Among various candidates as an alternative low-GWP precursor, the investigation into C_6_F_6_ and C_4_H_2_F_6_ is reported here. It should also be noted that CHF_3_, an H-containing fluorocarbon precursor, is normally used for nitride etching. To the best of our knowledge, more than one material are etched simultaneously in an etching process and specifically in dielectric etching, etching selectivity of nitride-to-oxide or vice versa is essential. We thus consider that evaluating the etching performance of HFC precursors, such as CHF_3,_ on SiO_2_ is quite necessary. Additionally, we also consider that an HFC precursor should be compared with another HFC precursor, not a perfluorocarbon precursor like C_4_F_8_, to reduce any complexity that might be induced by H atoms included in HFC precursors but not in PFC ones. A low-GWP candidate, C_4_H_2_F_6_, was therefore compared with CHF_3_ to determine whether C_4_H_2_F_6_ was appropriate as an alternative to CHF_3_ or not.

We employ scanning electron microscopy and plasma diagnostics to analyze the etching results. Ion bombardment energy is determined by measuring self-bias voltages, while densities of various radical species are obtained through quadrupole mass spectroscopy. The obtained results include a comprehensive comparison of the etch performance, encompassing etch rate and selectivity, between C_4_F_8_ and C_6_F_6_, as well as between CHF_3_ and C_4_H_2_F_6_. Furthermore, we evaluate the greenhouse gas emissions using a million metric tons of carbon dioxide equivalent, which demonstrates significantly lower emissions when substituting conventional precursors with the proposed alternatives. The experimental setup and procedure details are provided in the subsequent sections.

## 2. Experiment

### 2.1. Processing Chamber Setup

**Figure 1:**
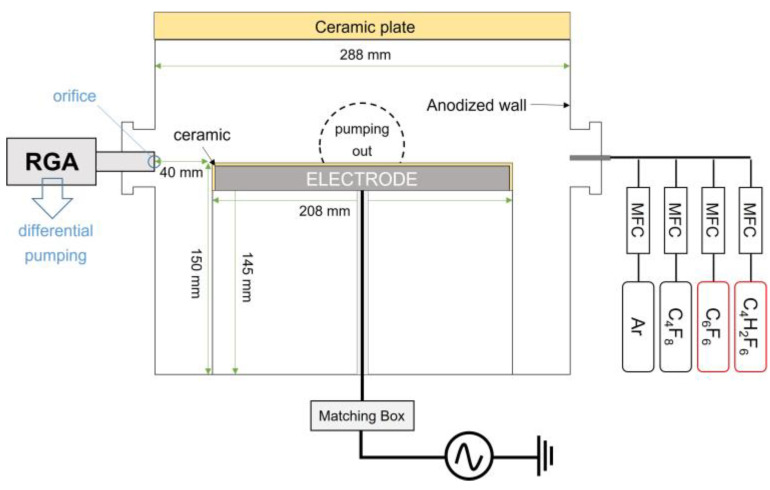
Schematic of the experimental setup.

The schematic diagram of the plasma chamber used for SiO_2_ etching is presented in Figure 1. Radiofrequency (RF) power of 13.56 MHz is applied to the electrode, which has a diameter of 208 mm covered with a 2 mm-thick ceramic plate, through an impedance matching box. While being injected into the chamber via a 1/4” SUS tube, the flow rate of Ar is controlled by a mass flow rate controller (MFC) (1179A611522K, MKS Instruments Inc., Andover, MA, USA) and that of PFC and HFC precursors is controlled by the different model of an MFC (M3030VA, Line-Tech, Daejeon, Republic of Korea). The investigated PFC precursors are octafluorocyclobutane (C_4_F_8_) and hexafluorobenzene (C_6_F_6_), while the investigated HFC precursors are trifluoromethane (CHF_3_) and hexafluoro-isobutylene (C_4_H_2_F_6_). The specific material information on the investigated precursors is summarized in Table 1.

The flow rate of Ar and a fluorocarbon precursor are fixed at 20 sccm and 10 sccm, respectively. It should be noted that the canisters containing C_6_F_6_ and C_4_H_2_F_6_ are heated to 130 °C in order to vaporize them, and are in the liquid phase at room temperature. The RF power applied ranges from 300 W to 500 W and the pressure varies from 10 mTorr to 30 mTorr (Table 2). When the RF power varies, the pressure is regulated by a throttle valve; when the pressure changes, the RF power is fixed at 400 W. The chamber is evacuated by a turbomolecular pump, with an oil rotary pump serving as a backing pump, to achieve a base pressure below 10^−4^ Torr.

### 2.2. Plasma Diagnosis

We utilized a high-voltage (HV) probe (P5100, Tektronix, Inc., Beaverton, OR, USA) and a residual gas analyzer (RGA) (SRS-200, Stanford Research Systems, Sunnyvale, CA, USA) to analyze the SiO_2_ etching results obtained with different precursors. The HV probe is connected to the transmission line responsible for RF input power positioned between the electrode and the matching box. This measures the self-bias voltage of the electrode, providing an estimation of the ion bombarding energy during plasma processing [32]. RGAs are widely employed plasma diagnostics tools that offer information about gas-phase species in plasmas [33,34,35,36]. With an RGA, gas-phase atoms or molecules ionized by the collision with 70 eV energetic electrons and then filtered based on their mass as they pass through a quadrupole mass filter operating in a cross-linked manner [37]. This filtering process enables the RGA to generate a signal intensity profile as a function of atomic mass [38]. The RGA, located on a chamber port, has a separate vacuum space from the main chamber via an orifice with the diameter of approximately 100 μm, which helps in maintaining the RGA pressure at approximately two orders of magnitude lower than the main chamber pressure [39]. The orifice is considered to directly contact to the plasma diagnosed as the chamber wall. This dedicated vacuum system allows gas-phase atoms and molecules to traverse the RGA without experiencing collisions with background gas, thereby increasing data reliability.

It should be noted that the impact of electrons of 70 eV would result in a measurement error from the dissociative ionization reaction of a parent molecule [40]; specifically, if C_4_F_8_ collides with 70 eV electrons, CF_2_^+^ ions can be generated and they are almost indistinguishable from the CF_2_^+^ ions generated in plasma in QMS analysis. Reducing the noise from dissociative ionization will be addressed in future work.

### 2.3. Sample Preparation

**Figure 2:**
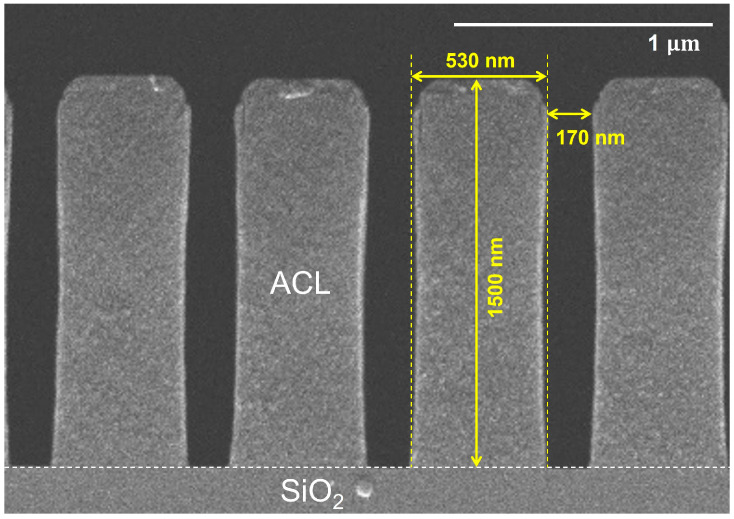
Reference SEM image of an un-etched SiO_2_ coupon wafer.

We prepared SiO_2_ coupon wafers with a masking layer of amorphous carbon (ACL) that has bar-type deep-trench patterns. Figure 2 presents the cross-sectional view of the mask pattern before etching with a width of 170 nm, a depth of 1500 nm, and a pitch of 530 nm. This cross-sectional image of the not-etched SiO_2_ sample serves as a reference for comparing the images of the samples after etching. All the images presented in this paper were acquired using a scanning electron microscope (SEM) (SU7000, Hitachi, Ltd., Tokyo, Japan).

## 3. Results and Discussion

### 3.1. SiO_2_ Etching with PFC Precursors (C_4_F_8_ and C_6_F_6_)

**Figure 3:**
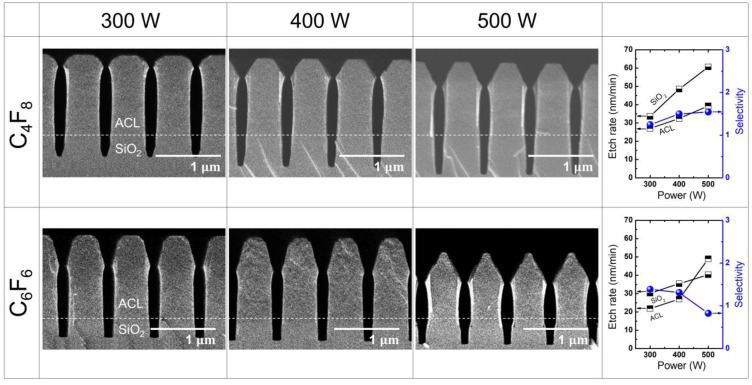
SEM images of the etch profile evolution with C_4_F_8_ and C_6_F_6_ at varying RF power from 300 W to 500 W. The pressure is maintained at 20 mTorr by controlling the Ar flow rate. The etch rate and selectivity with each precursor are plotted in the right-most column.

Figure 3 illustrates the etch results obtained with different PFC precursors (C_4_F_8_ and C_6_F_6_) at varying RF power levels ranging from 300 W to 500 W, while maintaining a fixed pressure of 20 mTorr. The resulting etch rates and selectivity for the C_4_F_8_ and C_6_F_6_ cases are summarized in the right-most column of Figure 3. The etch rate of ACL is calculated by measuring the difference between the lengths from the ACL-SiO_2_ border line to the top surface of ACL before and after etching, divided by the etching duration of 10 min. Similarly, the etch rate of SiO_2_ is determined by dividing the etch depth, defined as the length between the border line and the minimum position of the etched SiO_2_ trenches, by the etching duration of 10 min. The etch selectivity is then obtained by dividing the SiO_2_ etch rate by the ACL etch rate.

Regarding the SiO_2_ etch rate, there is no significant difference observed between C_4_F_8_ and C_6_F_6_, although the difference becomes more pronounced with increasing RF power. It is noteworthy that, despite the significantly higher GWP_100_ of C_4_F_8_ (9540) compared with C_6_F_6_ (7) by over 1000 times [23], the similar etch rate implies that adopting the low-GWP alternative precursor can result in a substantial reduction in the greenhouse effect without compromising etch throughput.

As depicted in Figure 3, it can be observed that the SiO_2_ and ACL etch rates with C_4_F_8_ increase almost linearly with increasing RF power, leading to a nearly constant selectivity. On the other hand, with C_6_F_6_, it is notable that the ACL etch rate abruptly increases with increasing RF power while the SiO_2_ etch rate shows a slight linear increase. Consequently, this rapid increase in ACL etch rate causes the selectivity to drop below 1. Such low selectivity with C_6_F_6_ can be attributed to the mask faceting, which worsens with higher RF power. Comparing the etch profiles obtained with C_4_F_8_ and C_6_F_6_, it is evident that mask faceting is more pronounced with C_6_F_6_ than with C_4_F_8_. For both C_4_F_8_ and C_6_F_6_ cases, it is commonly observed that, as RF power increases, mask faceting tends to deteriorate. This deterioration results in bowing of the mask, referring to a concave etch profile caused by the lateral, as well as vertical etching of the trench sidewall due to ion bombardment on the facet [3]. According to material sputtering theory, the sputtering yield is highest at incident angles ranging from 40° to 60°, rather than 90° [41]. This behavior is clearly reflected in the etch results shown in Figure 3. Comparing the transition of mask height and faceting, it is observed that, while the mask height barely changes, the mask faceting evolves rapidly with increasing RF power. Mask faceting induces mask bowing, which eventually affects the SiO_2_ etch profile as the concave mask profile expands across the mask-SiO_2_ border. Such mask faceting occurs more intensely with C_6_F_6_ than with C_4_F_8_, as presented in Figure 3. Notably, an increase in RF power from 400 W to 500 W resulted in a significant rise in the mask etch rate. This could be attributed to the left and right mask facets meeting during etching, forming a spear-like mask head. As discussed earlier, ion bombardment is more effective on a tilted surface compared with a flat surface, and thus the spear-like mask head created by the joint of the left and right facets could lead to the rapid mask etching.

To analyze the differences in etching behavior between C_4_F_8_ and C_6_F_6_ precursors, we investigated changes in ion bombardment energy and radical density. In a capacitively coupled plasma etching system, such as the one used in this work, where the samples are loaded on the bottom electrode, also as in this work, the powered electrode (bottom electrode) voltage primarily consists of a negative DC component that accelerates the bombarding ions, allowing them to reach even the bottom of deep trenches [41]. Therefore, it is crucial to measure the self-bias voltage of the powered electrode to determine the ion bombardment energy under specific plasma conditions. We employed a high-voltage probe to measure the self-bias voltage on the powered electrode.

**Figure 4:**
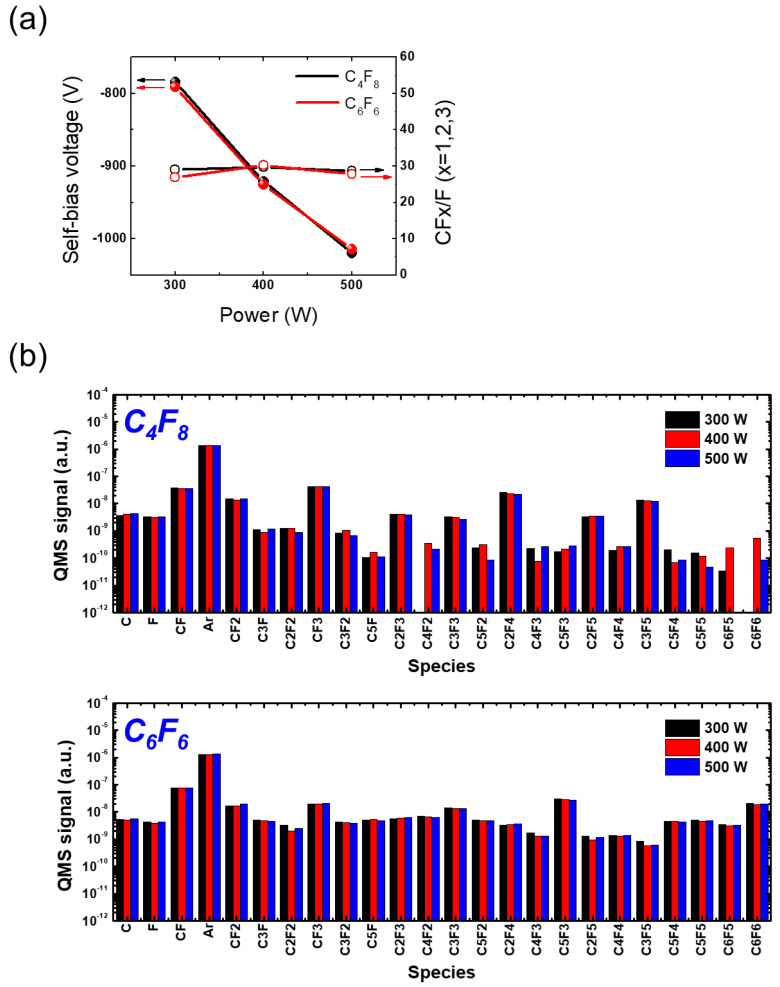
(**a**) Self-bias voltage and (**b**) radical species signal at varying RF power with PFC gas mixtures of C_4_F_8_ and C_6_F_6_. The pressure is maintained at 20 mTorr.

Figure 4a shows the measured self-bias voltage (left *y*-axis) and the CFx/F (x = 1, 2, 3) ratio (right *y*-axis) as a function of applied RF power using C_4_F_8_ and C_6_F_6_. The CFx/F ratios are calculated by using the FC radical signals represented in Figure 4b where CFx refers to the sum of the CF, CF_2_, and CF_3_ signal and is divided by the F signal at different RF power conditions. Note that the parameter, a CFx/F ratio, is adopted to interpret the chaotic contribution of numerous radical species to etch results in a simple manner. More detailed analysis of the measured radical densities will be followed separately. It can be observed that, as the RF power increases, the absolute value of the self-bias voltage increases, indicating an increase in ion bombardment energy. Notably, there is little difference between the self-bias voltages with C_4_F_8_ and C_6_F_6_, suggesting that the ion bombardment energy is very similar for both precursors. Furthermore, the absolute value of a self-bias voltage is known to be proportional to the plasma electron density. In CCP at a constant RF power, the dominant current path in the bulk plasma is the conduction current by plasma electrons so that, if the electron density decreases, the voltage between plasma and the electrode increases to meet the constant input power, a product of current and voltage [41]. In this context, the almost identical self-bias voltages at different RF powers may indicate that the ion density arriving at the etched surface is also very similar. In this case, the etch results are primarily influenced by the density of radical species [42]. As shown in Figure 4a, the CFx/F ratios at different RF powers are higher with C_4_F_8_ than C_6_F_6_, which indicates that polymerization occurs more with C_4_F_8_ than C_6_F_6_. This might lead to the better passivation of trench sidewalls during etching with C_4_F_8_, as shown in Figure 3.

In plasma etching, the synergetic effect between ions and radicals plays a significant role [43]. Therefore, measuring the radical composition is just as essential as investigating the ion bombardment energy. Radicals are generated through the dissociation of parent molecules by electron impact in plasma etching. Complex molecules like C_4_F_8_ undergo dissociation into various species with a wide range of molecular masses, including light molecules, such as CF and CF_2_, as well as heavy molecules, such as C_2_F_3_ and C_3_F_5_ [44]. This wide range of species has led to the extensive use of mass spectroscopy for investigating FC plasma chemistry [38].

As depicted in Figure 4b, while low-mass radicals, such as F, CF, and CF_2_, show no significant dependence on the power variation, a notable difference in high-mass radical signal between C_4_F_8_ and C_6_F_6_ is observed. Several high-mass species, such as C_2_F_4_, C_2_F_5_, and C_3_F_5_, exhibit higher densities with C_4_F_8_ compared with C_6_F_6_, primarily due to the dominant dissociation pathways of C_4_F_8_ in plasma [44]. Apart from these radicals, high-mass species are observed to be predominantly generated through the dissociation of C_6_F_6_. This may contribute to intense faceting of the ACL mask during etching with C_6_F_6_ by inducing the creation of high-mass ions that could transfer larger momentum to the ACL mask compared with low-mass species ions. The identification of the existence of such high-mass ions, however, is not conducted in this work due to the unverified ion measurement ability of the QMS equipment used. The modification of the equipment for reliability ion density measurement will be addressed in future work.

**Figure 5:**
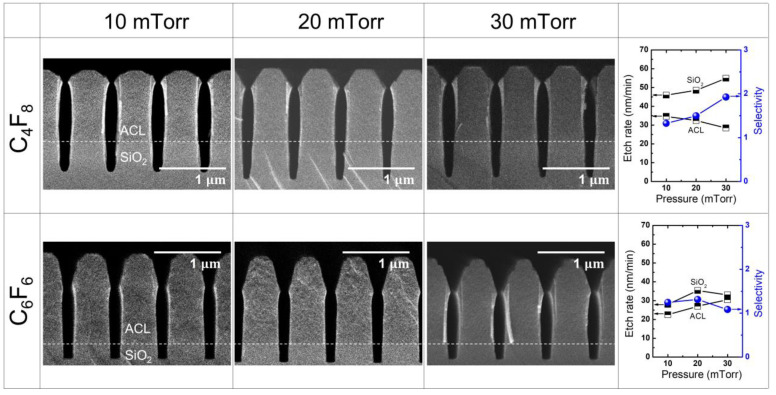
SEM images of the etch profile evolution with C_4_F_8_ and C_6_F_6_ at varying pressure from 10 mTorr to 30 mTorr. The RF power is maintained at 400 W. The etch rate and selectivity with each precursor are plotted in the right-most column.

Figure 5 illustrates the etch results obtained with different PFC precursors (C_4_F_8_ and C_6_F_6_) at varying pressure ranging from 10 mTorr to 30 mTorr, while maintaining a fixed RF power of 400 W. The resulting etch rate and selectivity for the C_4_F_8_ and C_6_F_6_ cases are summarized in the right-most column of Figure 5. When etching with C_4_F_8_, a notable trend is observed where the SiO_2_ etch rate increases with increasing pressure, while the ACL etch rate decreases. As a result, there is a significant increase in selectivity. Conversely, when using C_6_F_6_, the SiO_2_ etch rate initially rises with increasing pressure but eventually starts to decrease at higher pressure, while the ACL etch rate shows a linear increase. Consequently, this leads to a decrease in selectivity. These findings suggest that the optimal SiO_2_ etching window for C_6_F_6_ exists at lower pressures than that of C_4_F_8_, as the etch rate and selectivity with C_6_F_6_ can be comparable to those achieved with C_4_F_8_.

Regarding mask faceting, the etch profiles of the mask with both C_4_F_8_ and C_6_F_6_ show a relatively constant extent of faceting as the pressure increases. This is in contrast to the changes observed with variations in RF power, as depicted in Figure 3. This characteristic of mask faceting is advantageous for SiO_2_ etching, as it induces rapid etching of the mask and leads to bowing in the etch profile of both the mask and the substrate.

**Figure 6:**
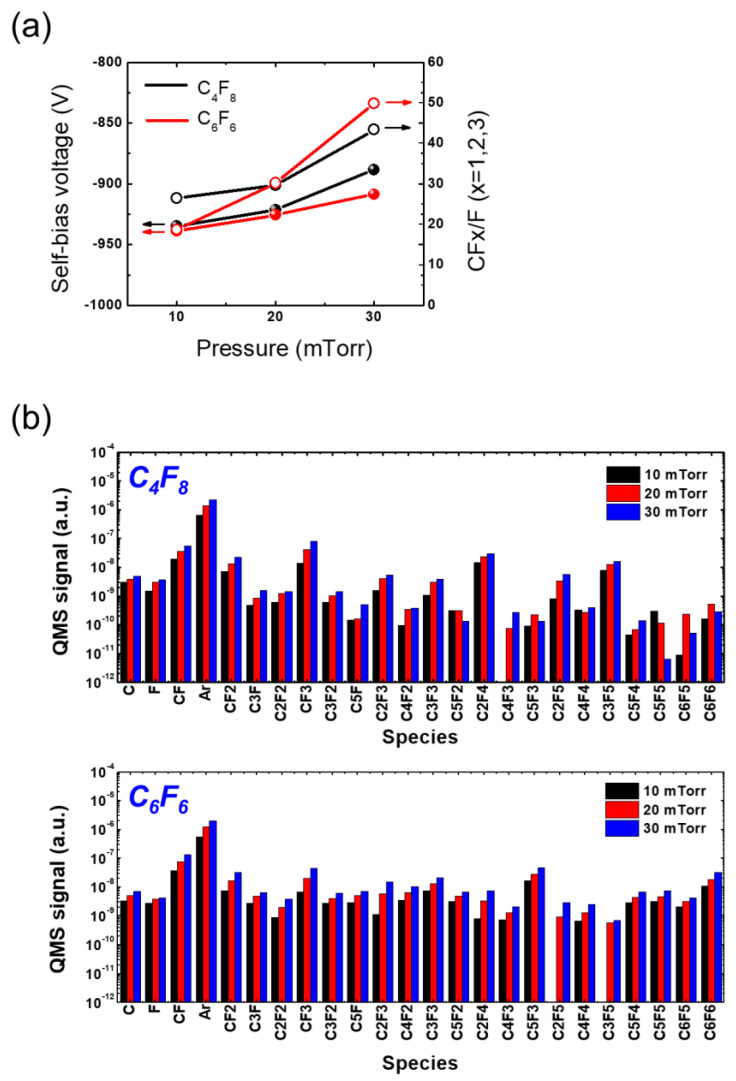
(**a**) Self-bias voltage and (**b**) radical species signal at varying pressure with PFC gas mixtures of C_4_F_8_ and C_6_F_6_. The RF power is maintained at 400 W.

Figure 6a illustrates the relationship between the measured self-bias voltage (left *y*-axis) and the CFx/F (x = 1, 2, 3) ratio (right *y*-axis) and pressure with C_4_F_8_ and C_6_F_6_. As the pressure increases, the self-bias voltage also increases, indicating a decrease in ion bombardment energy. With the change in pressure from 10 mTorr to 20 mTorr, the self-bias voltages show minimal difference between C_4_F_8_ and C_6_F_6_. However, as the pressure continues to rise, the disparity in self-bias voltage between C_4_F_8_ than C_6_F_6_ becomes more pronounced, with C_4_F_8_ exhibiting a larger increase. Since the absolute value of the self-bias voltage with C_4_F_8_ at 30 mTorr is lower than that with C_6_F_6_, the ion density and energy reaching the substrate are considered to be less, compared with the C_6_F_6_ case. This finding will aid in the interpretation of the etching discrepancy observed with C_4_F_8_ and C_6_F_6,_ along with measurements of radical species.

Figure 6b demonstrates the changes in radical species signal as pressure increases for C_4_F_8_ and C_6_F_6_. As the pressure increases, the F signal is found to increase for both cases. However, the SiO_2_ etch rate with C_6_F_6_ does not follow the F signal trend regarding the pressure change, which implies that the contribution of radical species to etching should be addressed through comprehensive consideration of various radical species. There is a significant disparity in high-mass radical signal between C_4_F_8_ and C_6_F_6_, as observed in Figure 4b. Additionally, the dependence of radical species signal on pressure is much substantial compared with its dependence on RF power.

Considering the slight decrease in self-bias voltage and the simultaneous rapid increase in radical species signal with varying pressure, it is expected that the SiO_2_ etch rate would decrease as the deposition rate increases more than the etch rate [42]. However, contrary to this expectation, Figure 5 reveals that the SiO_2_ etch rate with C_4_F_8_ actually increases as the pressure rises. This anomalous behavior can be attributed to the increasing difficulty of radical transport toward the trench bottom as the trench’s aspect ratio rises. Since a SiO_2_ etch rate increases when the depositing FC film is very thin and eventually decreases and saturates when the FC film becomes significantly thick [45], the monotonic increase in the SiO_2_ etch rate with C_4_F_8_ depicted in Figure 5 appears to be caused by the insufficient radical supply [46].

Overall, a comparison of ACL-masked SiO_2_ etch results with C_4_F_8_ and C_6_F_6_ under variations in RF power and pressure reveals that C_6_F_6_ exhibits superior etch rate and selectivity. This finding is even more remarkable considering that C_6_F_6_ has a GWP_100_ over 1000 times lower than that of C_4_F_8_. While C_6_F_6_ demonstrates a nearly identical self-bias voltage to that of C_4_F_8_, indicating similar ion bombardment energy, the plasma radical chemistry significantly differs. However, ACL etching with C_6_F_6_ experiences mask faceting, leading to rapid mask etching and decreased selectivity. Thus, it is necessary to identify a more optimal etching window for C_6_F_6_ to enhance performance for both ACL and SiO_2_ etching processes.

### 3.2. SiO_2_ Etching with HFC Precursors (CHF_3_ and C_4_H_2_F_6_)

**Figure 7:**
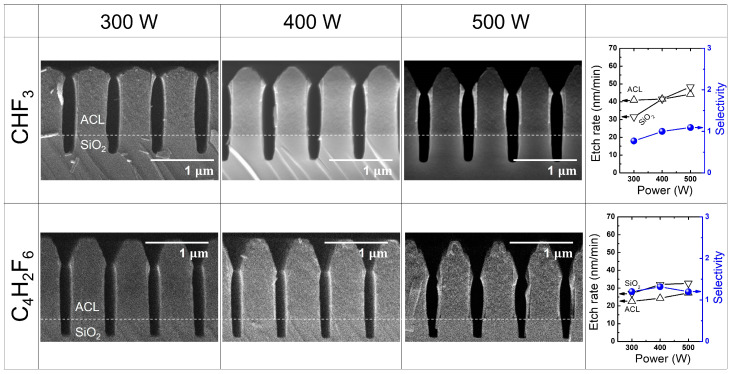
SEM images of the etch profile evolution with CHF_3_ and C_4_H_2_F_6_ at varying RF power from 300 W to 500 W. The pressure is maintained at 20 mTorr by controlling the Ar flow rate. The etch rate and selectivity with each precursor are plotted in the right-most column.

Figure 7 presents the etch results obtained with different HFC precursors (CHF_3_ and C_4_H_2_F_6_) at varying RF power levels from 300 W to 500 W, while maintaining a fixed pressure of 20 mTorr. The resulting etch rates and selectivity values for C_4_F_8_ and C_6_F_6_ are summarized in the right-most column of Figure 7. It is commonly observed that as RF power increases, mask faceting tends to deteriorate, as evidenced in the etch results with C_4_F_8_ and C_6_F_6_. While the use of CHF_3_ produces nearly vertical ACL etch profiles, the utilization of C_4_H_2_F_6_ at an RF power of 500 W results in a distorted ACL etch profile. This distortion appears to be induced by the rough mask surface, which causes random ion scattering. However, when the RF power is set below 500 W, sharp and vertical trench etching can be achieved without any defects, such as bowing.

Regarding the etch rate and selectivity, CHF_3_ exhibits a SiO_2_ etch rate lower than that of ACL. As RF power increases, this trend is reversed, leading to a selectivity slightly above unity. On the other hand, with C_4_H_2_F_6_, both the SiO_2_ and ACL etch rates gradually increase as RF power increases. The etch rate of SiO_2_ remains slightly higher than that of ACL, while the selectivity remains above unity, even at an RF power of 500 W when the mask sidewall is distorted, possibly due to random ion scattering. Although the SiO_2_ etch rate with C_4_H_2_F_6_ is slightly lower than that with CHF_3_, the use of C_4_H_2_F_6_ yields a comparable etch selectivity with that of CHF_3_. Additionally, with minor optimization of the processing pressure, such as decreasing the pressure as demonstrated in Figure 7b, a fine etch profile could be achieved.

**Figure 8:**
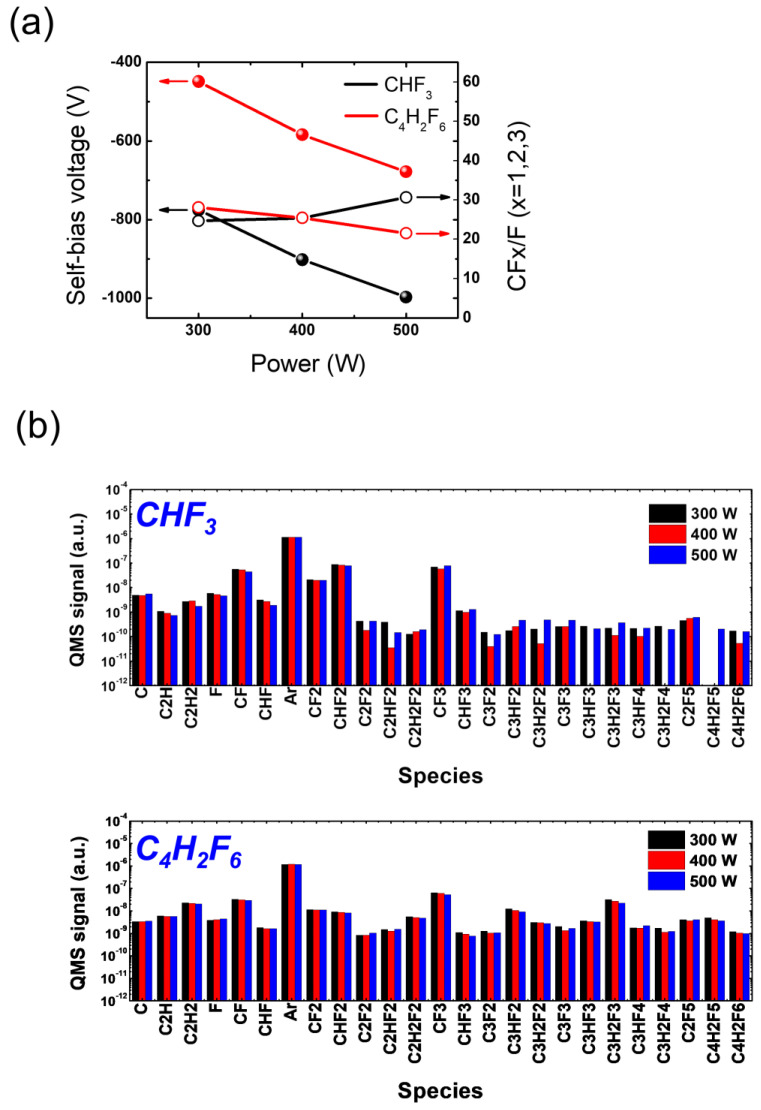
(**a**) Self-bias voltage and (**b**) radical species signal at varying RF power with HFC gas mixtures of CHF_3_ and C_4_H_2_F_6_. The pressure is maintained at 20 mTorr.

Figure 8a depicts the measured self-bias voltage (left *y*-axis) and the CFx/F (x = 1, 2, 3) ratio (right *y*-axis) as a function of applied RF power using CHF_3_ and C_4_H_2_F_6_. As the RF power increases, both CHF_3_ and C_4_H_2_F_6_ show an increase in the absolute value of the self-bias voltages, indicating an increase in the ion bombardment energy. Notably, the absolute value of the self-bias voltages with CHF_3_ at different RF powers is approximately twice that with C_4_H_2_F_6_, indicating more intense ion bombardment during etching with CHF_3_ compared with C_4_H_2_F_6_. This higher ion energy can be advantageous for HARC etching, where sufficient ion energy is required to reach the trench bottom. Etching with C_4_H_2_F_6_ would therefore require additional biasing to enhance the ion bombardment in order to match that achieved with CHF_3_.

Figure 8b presents the signal of various radical species resulting from the dissociation of CHF_3_ and C_4_H_2_F_6_, which varies with increasing RF power. As the RF power increases, CHF_3_ and C_4_H_2_F_6_ show different trends of radical signal variation species by species. Specifically, F from CHF_3_ decreases with increasing RF power, while that from C_4_H_2_F_6_ increases slightly. Those trends, however, do not follow the etch rate trend that changes with an increase in RF power, which might be attributed to a complex contribution of other FC radicals to the etch rate. When comparing CHF_3_ and C_4_H_2_F_6_, it is noteworthy that C_4_H_2_F_6_ yields carbon-rich radical species that contain multiple carbon atoms in their chemical structure, while CHF_3_ generates them to a lesser extent. This difference is expected since the generation of such carbon-rich radicals from CHF_3_ dissociation requires additional recombination processes between carbon-containing species such as CF_2_ and CHF_2_, which have relatively low reaction rates. However, this particular behavior does not appear to have a significant impact on ACL-masked SiO_2_ etching. Despite the prevalence of carbon-rich radicals in the process, the use of C_4_H_2_F_6_, as shown in Figure 7, does not demonstrate substantial advantages in terms of ACL mask protection or SiO_2_ trench profiles.

Nevertheless, it may be meaningful to focus on the difference in radical compositions between CHF_3_ and C_4_H_2_F_6_, particularly in relation to the major dissociation fractions of CHF_3_, rather than solely considering the evident difference in plasma chemistry between the two gases. This is because the major species resulting from CHF_3_ dissociation could play a critical role in determining the etch results. In Figure 8b, it can be observed that C_4_H_2_F_6_ dissociation yields comparable densities for most major species compared with CHF_3_ fractions. However, the densities of C_2_H_2_ and CHF_2_ are significantly lower with C_4_H_2_F_6_ than with CHF_3_. Furthermore, it is found that the densities of major species are barely affected by RF power variation, suggesting the need for another control parameter to manipulate plasma conditions.

**Figure 9:**
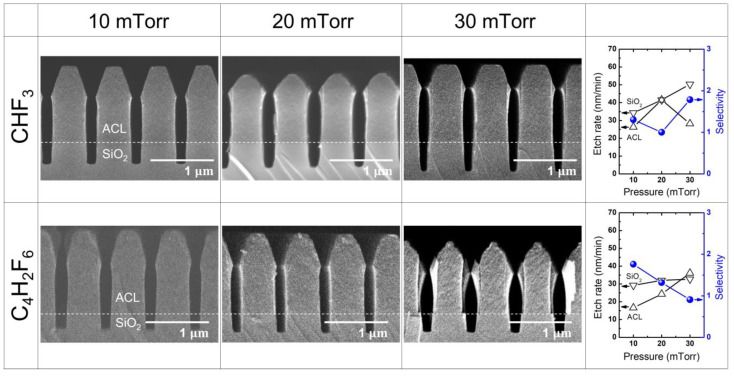
SEM images of the etch profile evolution with CHF_3_ and C_4_H_2_F_6_ at varying pressure from 10 mTorr to 30 mTorr. The RF power is maintained at 400 W. The etch rate and selectivity with each precursor are plotted in the right-most column.

Figure 9 illustrates the etch results obtained with different HFC precursors (CHF_3_ and C_4_H_2_F_6_) at varying pressures from 10 mTorr to 30 mTorr, with a fixed RF power of 400 W. The resulting etch rate and selectivity for the CHF_3_ and C_4_H_2_F_6_ cases are summarized in the right-most column of Figure 9.

When etching with CHF_3_, an interesting trend is observed. The ACL etch rate initially decreases and then rebounds as the mask faceting reduces, while the SiO_2_ etch rate monotonically increases. This behavior leads to a minimum selectivity at the intermediate pressure of 20 mTorr. In contrast, when using C_4_H_2_F_6_, ACL etching continuously increases with enhanced mask faceting as the pressure rises, resulting in significant bowing at the mask sidewall. At the same time, SiO_2_ etch rate shows minimal change with increasing pressure, leading to a substantial decrease in etch selectivity.

These findings highlight the importance of pressure control in achieving desirable etch results. With CHF_3_, an optimal pressure range can be identified to balance ACL etch rate and SiO_2_ etch rate, thereby maximizing selectivity. On the other hand, C_4_H_2_F_6_ exhibits challenges in maintaining selectivity as pressure increases, leading to distorted mask sidewalls and reduced etch performance.

Overall, understanding the pressure-dependent behavior of different HFC precursors is crucial for optimizing the etching process and achieving the desired etch profiles and selectivity.

**Figure 10:**
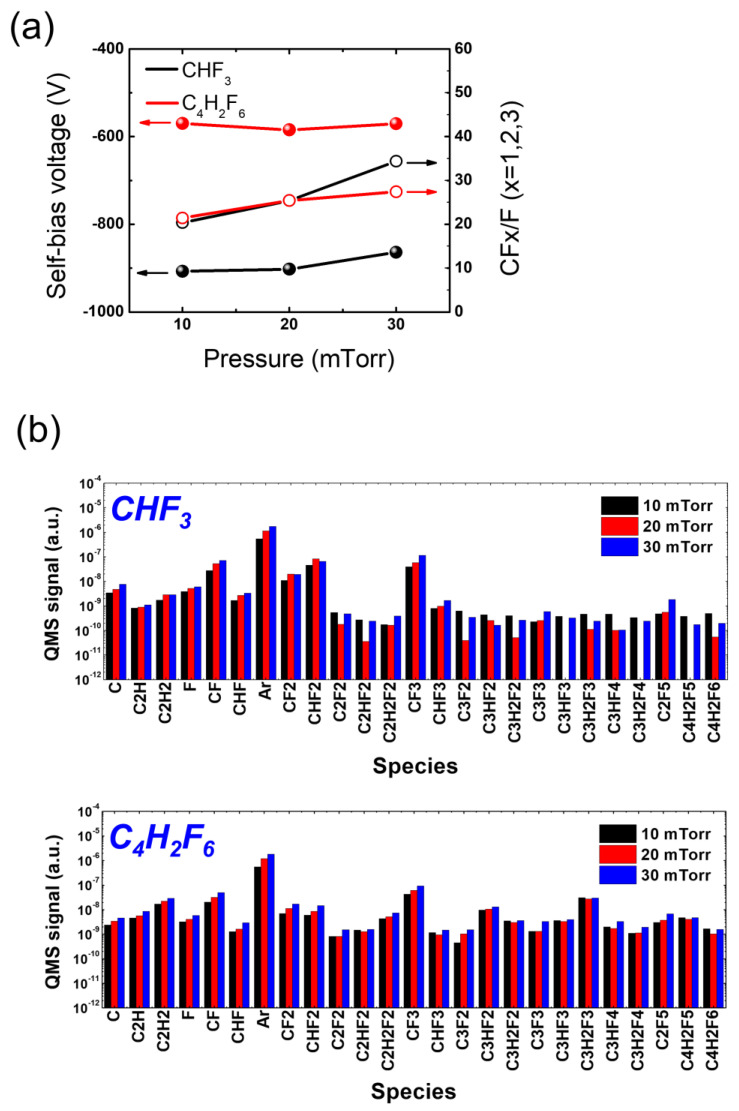
(**a**) Self-bias voltage and (**b**) radical species signal at varying pressure with HFC gas mixtures of CHF_3_ and C_4_H_2_F_6_. The RF power is maintained at 400 W.

Figure 10a illustrates the measured self-bias voltage (left *y*-axis) and the CFx/F (x = 1, 2, 3) ratio (right *y*-axis) as a function of pressure for CHF_3_ and C_4_H_2_F_6_. It is observed that with increasing pressure, both CHF_3_ and C_4_H_2_F_6_ show no significant change in the self-bias voltages. However, it is important to note that CHF_3_ consistently provides higher absolute self-bias voltages compared with C_4_H_2_F_6_ across a wide range of pressures. This indicates the ion bombardment during etching is stronger with CHF_3_ than with C_4_H_2_F_6_ owing to higher ion density and energy. This difference in ion bombardment strength may be linked to the higher SiO_2_ etch rate observed with CHF_3_ compared with C_4_H_2_F_6_.

In Figure 10b, the signal of various radical species resulting from the dissociation of CHF_3_ and C_4_H_2_F_6_ is presented as a function of increasing pressure. As previously observed in Figure 8b, carbon-rich radical species containing multiple carbon atoms in their chemical structure are significantly observed with C_4_H_2_F_6_, while they are barely present with CHF_3_. Such carbon-rich species are believed to react with ACL, enhancing its etching rather than passivating the ACL surface. Additionally, the key etchants of FC plasmas, CF_2_ and CHF_2_, are observed to exhibit reversed relative densities depending on the precursor used. In the case of CHF_3,_ the CF_2_ signal is lower than that of CHF_2_, whereas CF_2_ exceeds CHF_2_ with C_4_H_2_F_6_. This reversal in CF_2_ and CHF_2_ densities between the two precursors would be useful in selective etching of SiO_2_ over Si_3_N_4_ [47].

### 3.3. Evaluation of the Greenhouse Effect

Overall, our results demonstrate that the conventional FC gases of C_4_F_8_ and CHF_3_ exhibit a more vertical etch profile and a higher SiO_2_ etch rate across different power and pressure conditions. On the other hand, the alternative gases of C_6_F_6_ and C_4_H_2_F_6_ exhibit improved etch selectivity. However, when evaluating the etch performance of low GWP gases, it is crucial to assess the reduction in GHG emissions achieved by their implementation.

To quantify the environmental impact, we employ the million metric tons of carbon dioxide equivalents (MMTCDE) metric, which allows for a comparison of GHG emissions based on their GWP. This metric is particularly useful when a GHG is emitted in significantly smaller quantities compared with the reference amount of 1 ton. The carbon dioxide equivalent for a gas is derived as below.

(1) $$MMTCDE=\frac{Q\times GWP_{100}}{10^{9}}$$

In Equation (1), *Q* refers to the mass of a GHG emitted, measured in kg. To calculate *Q* for each PFC or HFC gas, we multiply the flow rate of the FC gases used for etching (10 sccm) by the processing time (10 min) to obtain the volumetric amount of the gas used. This value is then converted to mass in kg, taking into account the atomic mass unit of the gas. It should be noted that, in calculating the MMTCDE, we employ the mass of mother gas species, such as C_4_F_8_ and CHF_3_, rather than their fractions, including CF and CF_2_, that are observed by the RGA. This is based on a presumption that such reactive species would have relatively short lifetimes and thus have less affect global warming at the atmosphere compared with the chemically stable non-reactive molecules.

**Figure 11:**
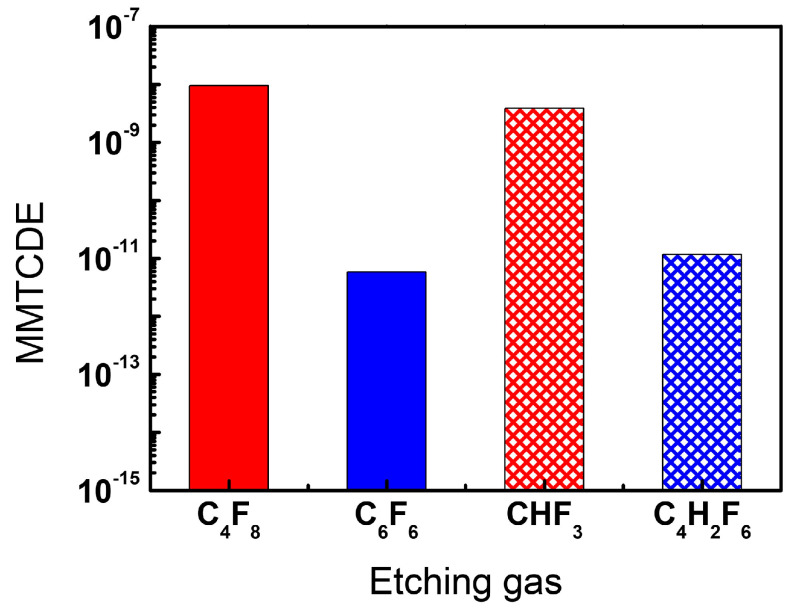
MMTCDE of the investigated etching gases during the etch process under the condition of 400 W RF power, 20 mTorr pressure, and 10 min duration.

Figure 11 illustrates a comparison of the estimated MMTCDEs for the conventional gases (C_4_F_8_ and CHF_3_) and the alternative gases (C_6_F_6_ and C_4_H_2_F_6_). The MMTCDE comparisons under the other experimental conditions show a nearly identical result so that we introduce only the result under etching conditions of 20 mTorr pressure and 400 W RF power for succinctness. Notably, the MMTCDEs associated with the alternative gases are approximately three orders of magnitude lower than those with the conventional gases, despite the etch results showing little difference. This observation suggests that the investigated alternative gases, C_6_F_6_ and C_4_H_2_F_6_, hold significant promise as candidates for reducing GHG emissions in the semiconductor industry.

It should be noted, however, that the above MMTCDE evaluation may not be sufficient since it is conducted only in the processing chamber, not at the exhaust line of the chamber. Even though low-GWP precursors such as C_6_F_6_ and C_4_H_2_F_6_, which are investigated in this work, are utilized in plasma processes, undesired high-GWP molecules such as C_2_F_6_ with 11,000 GWP can be created and exhausted into the atmosphere, which may reduce the benefits from the low-GWP precursors employment. Additionally, the MMTCDE evaluation with QMS could also include a certain level of inaccuracy due to the dissociative ionization by high-energy electron impacts. Thus, evaluating MMTCDE at the exhaust line of the vacuum chamber with non-invasive diagnostic tools would lead to more rigorous analysis on the global warming effects.

## 4. Concluding Remarks

In this study, we have explored the etch performance of C_6_F_6_ and C_4_H_2_F_6_ as alternatives to the conventional etch gases, C_4_F_8_ and CHF_3_, respectively, with the goal of reducing GHG emissions during plasma etching. Given the significantly lower GWP of the alternative gases, it is encouraging to investigate whether they can deliver comparable etch performance to the conventional gases.

To evaluate their performance, we conducted etching experiments using gas mixtures of different FC gases under various conditions of RF power and pressure. The obtained results were carefully analyzed using SEM images. Specifically, we compared the etch results of C_4_F_8_ with those of C_6_F_6_, and the etch results of CHF_3_ with those of C_4_H_2_F_6_.

Our findings indicate that the alternative gases demonstrated comparable etch performance to the conventional gases across various conditions. This suggests that the alternative gases hold promise for utilization in the next-generation etch processes with reduced GHG emissions. Importantly, our MMTCDE estimation revealed that replacing the conventional gases with the alternative gases could result in GHG emissions reduction by several orders of magnitude.

The outcomes of this study are expected to contribute to the wider adoption of low-GWP gases in semiconductor manufacturing. By incorporating these alternative gases into the etch processes, the industry can effectively reduce its environmental impact while maintaining the desired etch performance. Further research and development efforts should be directed towards optimizing the use of low-GWP gases and promoting their integration into commercial semiconductor fabrication processes.

## Figures and Tables

**Table 1:** The atmospheric lifetime and GWP100 of the investigated PFC and HFC precursors.

Name	Molecular Formula	Atmospheric Lifetime (Years)	GWP_100_	Reference
Octafluorocyclobutane	C_4_F_8_	3200	9540	[19]
Hexafluorobenzene	C_6_F_6_	0.23	7	[23]
Trifluoromethane	CHF_3_	222	12,400	[19]
Hexafluoro-isobutylene	C_4_H_2_F_6_	0.03	2.8	[31]

**Table 2:** The experimental conditions for the examined etching processes.

Power (W)	Pressure (mTorr)	Duration (min)	Gas Flow Rate (sccm)	Electrode Temperature (°C)
300–500	10–30	10	20 for Ar 10 for FC	10

## Data Availability

The data presented in this study are available on request from the corresponding author.
